# The Role of Cytokines in Cutaneous T Cell Lymphoma: A Focus on the State of the Art and Possible Therapeutic Targets

**DOI:** 10.3390/cells13070584

**Published:** 2024-03-28

**Authors:** Alba Guglielmo, Corrado Zengarini, Claudio Agostinelli, Giovanna Motta, Elena Sabattini, Alessandro Pileri

**Affiliations:** 1 Institute of Dermatology, Azienda Sanitaria Universitaria Friuli Centrale (ASUFC), 33100 Udine, Italy; 2 Dipartimento di Scienze Mediche e Chirurgiche, University of Bologna, 40138 Bologna, Italy; 3 Dermatology Unit, IRCCS Azienda Ospedaliero-Universitaria di Bologna, 40138 Bologna, Italy; 4 Haematopathology Unit, IRCCS Azienda Ospedaliero-Universitaria di Bologna, 40138 Bologna, Italy

**Keywords:** CTCL, lymphoma, cutaneous, skin, oncology, chemokine, inflammatory, mediators, protein

## Abstract

Cutaneous T cell lymphomas (CTCLs), encompassing mycosis fungoides (MF) and Sézary syndrome (SS), present a complex landscape influenced by cytokines and cellular responses. In this work, the intricate relationship between these inflammatory proteins and disease pathogenesis is examined, focusing on what is known at the clinical and therapeutic levels regarding the most well-known inflammatory mediators. An in-depth look is given to their possible alterations caused by novel immunomodulatory drugs and how they may alter disease progression. From this narrative review of the actual scientific landscape, Interferon-gamma (IFN-γ) emerges as a central player, demonstrating a dual role in both promoting and inhibiting cancer immunity, but the work navigates through all the major interleukins known in inflammatory environments. Immunotherapeutic perspectives are elucidated, highlighting the crucial role of the cutaneous microenvironment in shaping dysfunctional cell trafficking, antitumor immunity, and angiogenesis in MF, showcasing advancements in understanding and targeting the immune phenotype in CTCL. In summary, this manuscript aims to comprehensively explore the multifaceted aspects of CTCL, from the immunopathogenesis and cytokine dynamics centred around TNF-α and IFN-γ to evolving therapeutic modalities. Including all the major known and studied cytokines in this analysis broadens our understanding of the intricate interplay influencing CTCL, paving the way for improved management of this complex lymphoma.

## 1. Introduction

Cutaneous T cell lymphomas (CTCLs) are a group of non-Hodgkin lymphomas characterized by the appearance of the skin and the absence of extra-cutaneous dissemination at least six months after diagnosis [1,2]. Mycosis fungoides (MF) and Sézary syndrome (SS) are the two most common CTCLs, accounting for at least 50% of all CTCL diagnoses. From an epidemiological point of view, CTCL incidence has increased over recent decades, especially for MF and SS [3,4,5,6]. Hence, clinicians’ efforts are focused on understanding the mechanisms involved in CTCL pathogenesis to develop more effective and tailored therapies [7,8,9]. MF/SS pathogenesis and the mechanisms involved in their progression from early to advanced phases are far from being fully understood [10,11,12]. Different players are involved in MF/SS, such as gene alterations, changes in the microenvironment composition, cytokine balance changes, and transcriptional pathways [10,11,12,13,14,15,16]. All these players are intrinsically linked. 

The present paper aims to analyze the potential role of cytokine changes in MF/SS pathogenesis and progression and to investigate possible therapeutic targets by narratively reviewing the literature.

To list cytokines in the most concise but relevant manner, given the significantly changing panorama regarding the nomenclature, the number, and the definitions, we based our work on the papers of Dinarello [17] and Wautier et al. [18]. We then included the family of interleukins going from 1 to 33, tumor necrosis factor, interferons, platelets, fibroblasts, and epidermal growth factors.

For better readability of the text, a summary of the described cytokines, their mechanisms, and known and potential related therapies are listed in Table 1.

## 2. IL-1

The Interleukin 1 (IL-1) family includes many known pro-inflammatory cytokine subtypes that play crucial roles in amplifying the immune reaction and controlling various innate-response processes (IL-1β, IL-1α, IL-18, IL-33, IL-36α, IL-36β, IL-36γ, IL-36RA, IL-37, IL-38, and IL-1RA) [17,102]. IL-1 is also a known mediator of fever and a leukocytic endogenous cascade.

Nevertheless, in the tumoral environment, it behaves counterintuitively: in breast cancer, IL-1β is able to induce the recruitment of immunosuppressive cells such as tumor-associated macrophages (TAMs) and myeloid-derived suppressor cells (MDSCs), gaining advantages for neoplastic spread [102,103].

### IL-1 in MF/SS

IL-1′s role in MF/SS has been the subject of a few studies [104]. In these published works, it has been reported that low levels of immunostimulating cytokines (among them, IL-1 was included) are expressed in MF samples, speculating whether IL-1 may play a potential role in CTCL pathogenesis.

Another study showed how the major IL-1 subtypes alpha and beta are relatively less expressed in CTCLs when compared to healthy donors [19]. It has not been explained if this phenomenon has a pathogenetic role in halting disease progression, but it has been described as a potential biomarker in responsive patients to photopheresis [19,105].

## 3. IL-2

The physiological role of interleukin (IL)-2, also known as T cell growth factor (TCGF), is to upregulate the proliferation and cytolytic activity of activated T cells and to increase the cytotoxicity of monocytes and natural killer (NK) cells [21].

Thus, IL-2 is essential in activating the immune system against infective agents [21,106]. The IL-2 receptor (IL-2R) can be present in three subunits, IL-2Rα (also known as CD25), IL-2Rβ, and IL-2Rγ, and these are expressed on the surfaces of different cells important for activating memory T cells and prompting the immune response. IL-2Rγ (also known as common gamma chain or γc) dimerization is shared with the IL-4, IL-7, IL-9, IL-15, and IL-21 cytokines [44]. The interaction between IL-2 and IL-2R is a crucial stage of immune system activation. However, the IL-2Rα subunit plays a vital role in controlling and generating immune-suppressive T-regulatory cells (T-regs) [21]. IL-2 can act as an immunostimulating cytokine by expanding and increasing the proliferation of natural killer and cytotoxic CD8+ T cells. Moreover, IL-2 can induce the apoptosis of proliferating T cells through the Fas-FasL pathway and positively regulate T-reg cells, leading to immune tolerance and suppression [107].

### IL-2 in MF/SS

IL-2 has been the subject of some MF studies. Owing to IL-2’s ability to expand the immune response, several groups [108,109,110,111] have investigated the possible therapeutic role of a recombinant IL-2 in MF patients. However, contrasting results have been provided in the literature, and IL-2’s role in MF is still open. In 2001, Brockdorff et al. [22] highlighted the tumorigenic role of IL-2.

Indeed, the authors found high levels of Gab2, an adaptor molecule involved in IL-2 receptor signaling in CTCLs, via IL-2 stimulation; Brockdorff speculated whether Gab2 could be regarded as a possible MF/SS progression marker. However, the role of IL-2 and its receptor remains controversial in the literature.

Shohat et al. [23] hypothesized that IL-2R may have an immunosuppressive role rather than IL-2. By investigating the effect of AS101, a tellurium-based compound with immunomodulating properties, on the pattern of lymphokine production by peripheral blood mononuclear cells (PBMCs) from patients with mycosis fungoides, Shohat et al. [23] found that AS101 [24] inhibited the production of IL-2R, IL-5, and IL-10 and induced a significant increase in IL-2 levels in the mycosis fungoides PBMCs, suggesting that IL-2R may have an immunosuppressive role. In contrast, IL-2 may have an antitumor action.

Another potential drug targeting IL-2R is a fusion toxin called denileukin diftitox [112,113], a drug that intracellularly delivers diphtheria toxin and consecutively inhibits protein synthesis in IL-2R-expressing cells. In a murine model of IL-2R-expressing malignancies, denileukin diftitox prolonged survival compared with controls. The most common adverse events reported in patients who received denileukin diftitox were hypoalbuminemia, fever/chills, acute hypersensitivity reactions, nausea/vomiting, and asthenia.

Kaminetsky and Himes et al. used it in patients with IL-2R-positive cutaneous T cell lymphoma (CTCL), reaching an overall response of 36% [113].

Duvic et al. used the same drug in relapsed CTCLs in an open-label phase III trial with an overall response of 40%. However, progression-free survival was 205 days, the median duration of response was 274 days, and three patients withdrew because of toxicity. Still, these studies show how denileukin diftitox may provide a clinically meaningful response in CTCLs

Another potential drug targeting IL-2 signaling under development is CCR4-IL2 IT. IL2-CCR4 bispecific IT is a receptor-specific immunotoxin targeting both CCR4 and IL2Rα and has been shown in preclinical studies to be significantly more effective than IL2 fusion toxin and brentuximab [26]. Still, clinical trials are needed to assess its potential role in the treatment of human CD25+ and/or CCR4+ CTCL.

## 4. IL-3

Interleukin 3 (IL-3, also defined as multi-CSF), often in conjunction with other cytokines such as IL-5, regulates immune system activation against pathogens. Activated T cells and basophils secrete IL-3, fostering T cell growth and differentiation and stimulating macrophage and granulocyte maturation [27].

### IL-3 in MF/SS

To date, no studies have provided data on a role of IL-3 in MF/SS progression.

## 5. IL-4

Interleukin 4 (IL-4) is a cytokine involved in the skew to a Th2 phenotype of naïve T cells. Differentiation to a Th2 phenotype determines the consequence of a positive loop towards an increased production of IL-4 molecules. The receptor for interleukin-4 (IL-4Rα) has three different complexes throughout the body. Type 1, IL-4Rα, is effective when dimerized with the common gamma chain (γc), sharing the same mechanism as IL-2, IL-7, IL-9, IL-15, and IL-21 [27] and specifically binding IL-4. Type 2 receptors are bound to a different subunit, known as IL-13Rα1, and are shared with IL-12. One of the many physiological actions of IL-4 is to negatively modulate Th1 T cells, dendritic cells, and IFNγ production [114], facilitating tumor growth. 

On the other hand, it promotes Th2 T cell differentiation, promoting anti-inflammatory and immunosuppressive responses, specifically by promoting the recruitment and activation of immunosuppressive cells such as T-regs and MDSCs. In the literature, it is known that, in CTCLs, an imbalance towards Th2 polarization driven by IL-4 may contribute to immune evasion and tumor progression by suppressing anti-tumor immune responses and enhancing pro-tumorigenic microenvironments [115].

In brief, IL-4 exerts an immunosuppressive action and, from an oncologic point of view, acts as a tumorigenic cytokine [31,114,116]. 

### IL-4 in MF/SS

Since the early 1990s, increased production of IL-4 has been linked with CTCL progression [117,118,119,120]. IL-4 is thought to be one of the cytokines driving the shift from an antitumor to a tumorigenic phenotype. It decreases the production of Th1-associated cytokines, such as IL-12 and IFN-γ [121], so IL-4 can be regarded as an immunosuppressive molecule. IL-4, in association with IL-13, promotes tumor cell growth and proliferation in CTCL [122]. 

Furudate et al. hypothesized that IL-4 may act on the innate immune response by polarizing macrophages into immunosuppressive M2 macrophages. M2 macrophages produce Th-2 cytokines and stromal factors, promoting Th2 polarization of the microenvironment [123,124] 

IL-4 can also synergically act with IL-33, inducing the secretion of IL-31, an interleukin responsible for the pathogenesis of itch [84,125].

From another perspective, CTLCs are related to high levels of tumoral Th2 T cells, so blocking Th2 differentiation is thought to reduce or alter CTLC diseases. 

However, Saulite et al. asserted that a functional PD-1 block with nivolumab, in their pre-clinical work, resulted in a reduced Th2 phenotype of non-tumor T lymphocytes and enhanced the proliferation of tumor T cells in SS patients [32], shedding some light in the use of checkpoint inhibitors and CTCLs.

Still, blocking malignant T cells’ Th2 differentiation and proliferation by addressing the intracellular signaling pathway could be another feasible option. Ritlecitinib is a Janus kinase (JAK) inhibitor which has shown the capacity to reduce Th2 cells [126], and there is a phase IIA clinical trial evaluating this drug in CTCLs [33]. Guenova et al. [127] observed, in their analysis, that malignant T cells from patients with the disease typically express Th2 cytokines such as IL-4 and IL-13 but are negative for IFNγ, highlighting the MF cell’s ability to reduce immune system control by IFNγ low levels. High IL-4 levels also stimulate dermal fibroblasts from CTCL patients to secrete increased extracellular matrix protein periostin [128]. As a cascade of events, periostin will induce thymic stromal lymphopoietin expression, activating the STAT5 pathway and eventually promoting both neoplastic cell proliferation and the production of IL-4 [128,129,130,131]. A new monoclonal antibody against IL-4 (and IL-13), dupilumab, has recently become available for atopic dermatitis treatment [34]. 

Theoretically, by blocking IL-4 immune suppressive action, a reawakening of patients’ immune systems should be expected. However, in the literature, cases of misdiagnosed MF treated with dupilumab are available, featuring a dramatic disease progression [35,132,133]. It has been hypothesized that dupilumab action may paradoxically activate the JAK/STAT pathway, leading to results opposite to those expected.

## 6. IL-5

Interleukin 5 (IL-5) is a cytokine secreted by the activated Th-2 cell subset. IL-5 gene expression is linked with the IL-4 and IL-13 genes in a Th2 cytokine gene cluster on chromosome 5q [53].

The function of IL-5 has been best studied in the context of airway inflammation, particularly asthma. IL-5 promotes the differentiation, proliferation, recruitment, and activity of eosinophils, which are responsible for airway hyperreactivity and epithelial damage in allergic asthma [54,55]. 

### IL-5 in MF/SS

Eosinophilia and elevated levels of immunoglobulin E (IgE) are often observed in advanced CTCLs due to the shift to a Th2 response, and IL-5 represents the principal regulator of eosinophilia [134,135,136]. It has been assessed that IL-5 plays a crucial role in the development of eosinophilia in Hodgkin’s lymphoma. In CTCLs, the production of IL-5 has also been related to erythroderma and elevated serum levels of IgE [38]. It has been hypothesized that IL-5 may be overexpressed in CTCL, owing to the constitutive activation of the STAT3 pathway in neoplastic cells, while no evidence that non-malignant T cell lines may secrete IL-5 has been observed. [38] The potential immunosuppressive action of IL-5 was observed by Shohat et al. [23] by comparing the IL-2, IL-2R, IL-5, IL-10, and IFN gamma levels in the peripheral blood mononuclear cells (PBMCs) of healthy volunteers and CTCL patients. The Israeli group found significantly higher levels of IL-2R, IL-5, and IL-10 in the affected patients and significantly lower levels of interferon-gamma. After the use of AS101, a tellurium-based compound with immunomodulating properties, the cytokine profile changed, with an increase in IFN gamma and a decrease in immunosuppressive cytokines, evidence observed previously by Yamamoto et al. [39] after the administration of IFN gamma. 

Changes in IL-5 levels and other immunosuppressive or antitumor cytokines have been observed by other groups, highlighting a solid correlation between patient progression (and consequently, an increase in tumor burden both at the skin and blood levels) and an increase in immunosuppressive cytokine profile, including IL-5 [137]. In addition, other authors have proposed a correlation between IL-5 action and the miR-155/SATB1 axis, which is thought to play an essential role in CTCL progression and in other lymphoma pathogeneses. Low SATB1 expression levels, as observed in malignant SS cells, have been related to increased IL-5 production and higher malignant cell proliferation [51]. Aiming to add new possible response markers to scheduled therapies, Suchin at al. proposed that a decrease in IL-5 levels in treated patients may be regarded as a marker of clinical response to scheduled treatment, owing to the negative modulation of high antitumor cytokine levels such as IFN-α and IL-12 along with IL-5 [138]. 

## 7. IL-6

Interleukin 6 (IL-6) is a cytokine that synergistically acts in its role with IL-1. IL-6 induces acute-phase inflammation protein release, determines B lymphocyte differentiation into plasma cells, and increases adhesion molecules in inflammatory infiltrates [40,139].

### IL-6 in MF/SS

Since the 1990s, Lawlor et al. and Watson et al. [40,104] have provided evidence that IL-6 may induce lymphocyte activation and migration in MF samples. Lawlor et al. [104] observed high IL-6 levels in lesional samples, hypothesizing that IL-6 may be related to MF/SS pathogenesis. In their experiments, Watson et al. [78] observed that high T-lymphocyte concentrations in MF infiltrates were related to high IL-1 and IL-6 levels. The authors hypothesized that promoting lymphocyte adherence with IL-6 may be related to a selective modification in vascular adherence molecules. Kadin et al. [41] proposed a possible prognostic role of IL-6 in early MF patients. By analysing 96 early MF cases, the Authors found that high serum CD30, CD25, and IL-6 levels were related to a higher risk of MF progression. 

Olszewska et al. [43] recently proposed that the polymorphism of IL-6/STAT3 signaling may be related to the aggressive behavior of MF/SS. By comparing the serum IL-6 levels in 106 CTCL patients to 198 control cases, the authors found that their IL-6 serum levels were significantly higher than those in healthy controls. The same experiment also demonstrated that two genotypes, CC of IL-6 and GG of STAT3, were overexpressed in the CTCL patients, determining an increased risk of malignancy development. Due to the hypothesis that the GG genotype of the STAT3 polymorphism may be akin to the presence or absence of itch in CTCL patients, Olszewska et al. [43] highlighted that IL-6 may be involved in MF/SS, but not in the presence of itch in affected patients. 

Recently, IL-6 was been studied as a possible marker of therapeutic response after PUVA/nbUVB phototherapy by Karamova et al. [140]. The authors analysed lesional skin cytokine changes (IL-1β, IL-4, IL-6, IL-10, IL-17A, IL-17F, IL-21, IL-22, IL-23, IL-25, IL-31, IL-33, IFN-γ, sCD40L, and TNF-α) before and after the treatments, observing a positive correlation between an mSWAT decrease and the levels of IL22, IL33, and TNF-α in the tumor tissue. The levels of IL10 and IFN-γ after PUVA treatment were increased in comparison to baseline. However, no changes in IL-6 levels were found.

## 8. IL-7

Interleukin-7 (IL-7) is a hematopoietic growth factor secreted by stromal cells in the bone marrow and thymus [141]. It is also produced by keratinocytes, dendritic cells, hepatocytes, neurons, and epithelial cells, but not by normal lymphocytes [141].

It stimulates the development of lymphoid lineage by binding to its specific receptor and activating signal transduction when dimerized with the common gamma chain (γc), with the same mechanism as IL-2, IL-4, IL-9, IL-15, and IL-21 [44], activating intracellular signaling pathways through JAK/STAT that promote cell proliferation, survival, and differentiation (principally B cells, T cells, and NK cells) [45].

### IL-7 in MF/SS

In the medical literature, it has been written that an increased production of IL-7 could cause the chronic activation of CD8+ T cell clones in Sézary syndrome [142,143,144,145] and has been linked to the development of cutaneous lymphomas, given its relationship with lymphoid lineage progenitors’ stimulation.

However, there are very few works on the subject [142,144,146], and some have contrasting opinions, concluding that there is no in vivo confirmation of this correlation [46]. It is being studied as a potential immunotherapeutic drug [147], but there are no data regarding this use for CTCLs.

## 9. IL-8

See the paragraphs on Section 29.

## 10. IL-9

Interleukin 9 (IL-9) is a cytokine produced by various immune cells. It exerts its main activity when dimerized with the common gamma chain (γc), with the exact mechanism of IL-2, IL-4, IL-7, IL-15, and IL-21 on the IL-9 receptor [44], which is mainly expressed on T-helper cells subtype Th9, which are the major CD4+ T cells that produce IL-9. IL-33 can induce its production and also TGF-β [50].

Its primary function is to stimulate various hematopoietic cells’ proliferation and prevent the apoptosis of immune cells [50]. It activates the IL-9 receptor (IL9R) and intracellularly STAT pathway. It has also been linked to the development of asthma [148]. Due to its hematopoietic stimulations, it seems to give rise to multiple hematologic neoplasias and Hodgkin’s lymphoma, but it also has antitumoral properties in solid tumors, such as melanoma [149].

### IL-9 in MF/SS

It seems that its hematopoietic function may be related to the appearance and maintenance of lymphomatous neoplasms [50].

This has been confirmed in some studies in which increased levels of Il-9 have been linked to patients suffering from Sézary syndrome [51] and mycosis fungoides [52].

As with Il-7, interest has been shown in stimulating its receptor as immunotherapy; however, there are currently no active studies on the uptake of CTCLs [150].

## 11. IL-10

Interleukin 10 (IL-10) plays a central role in preventing autoimmune diseases. IL-10 negatively modulates immune response during infection, allergy, and autoimmunity by increasing immunosuppressive cell levels [151]. On the other hand, due to its intrinsic immunosuppressive activity, it has been hypothesized that IL-10 may contribute to chronic infections [152]. An autocrine loop also regulates the production of IL-10. 

Indeed, IL-10 is thought to be increased by T-regs lymphocytes recruited from blood vessels. IL-10 exerts its immunosuppressive action by inhibiting proinflammatory Th1 lymphocytes and the proliferation and differentiation of B and Th2 cells.

### IL-10 in MF/SS

Higher levels of IL-10 have been detected in MF/SS biopsies compared with normal skin, corroborating the hypothesis that IL-10 may be involved in MF/SS pathogenesis and progression. Unsurprisingly, in their experiments, Wu et al. observed that higher levels of IL-10 can be seen in advanced CTCL phases rather than early ones [153]. Similar evidence was observed by Akatsuka et al. [55], who found that the development of IL-10-producing Bregs is impaired in patients with advanced MF, and a decrease in IL-10-producing Bregs may play an essential role in the progression of advanced MF. Different groups have focused on IL-10 as a possible therapeutic target or marker. 

Tiffon et al. proposed that one of the histone deacetylase inhibitors, such as vorinostat and romidepsin, may exert their therapeutic action due to the downregulation of IL-10 RNA expression [56]. A similar action has been proposed for some proteasome inhibitors, like bortezomib, that modulate cytokine expression in CTCL, acting on TGFβ1 and IL-10 down-regulation [57]. IL-10′s role has also been studied from a genetic point of view, and upon reviewing the studies on the polymorphisms of the cytokine genes involved in CTCL pathogenesis, the paper by Nedoszytko B et al. is of some interest. Indeed, the authors observed that a polymorphic variant in the promoter region of IL-10 can reduce transcription factor recognition, leading to a decreased level of IL-10. The authors questioned whether such a gene variant may be related to a reduced risk of developing MF [154].

## 12. IL-11

Interleukin-11 (IL-11) plays a pivotal physiological role in multiple systems in the body. Primarily known for its involvement in hematopoiesis, it stimulates the production of platelets from megakaryocytes and hemostasis. Additionally, IL-11 showcases anti-inflammatory properties, modulating immune responses to maintain a balanced and regulated inflammatory environment [58].

### IL-11 in MF/SS

While research continues, no direct established link exists between IL-11, MF, or SS.

## 13. IL-12

Interleukin 12 (IL-12) is a heterodimeric cytokine encoded by two separate genes, IL-12A and IL-12B (in common with IL-23), mainly transduced and transcribed by antigen-presenting cells, such as macrophages and dendritic cells [155,156].

Being a heterodimeric protein makes it included, from a structural perspective, in a family with IL-23, IL-27, and IL-35 [59]. Despite this, it has a different biological function [59], which consists of activating natural killer (NK) cells and promoting the differentiation of naive T cells into Th1 cells, thereby enhancing the immune system’s ability to fight intracellular pathogens and regulate inflammatory responses [155].

Finally, it stimulates IFNγ production by T cells, which primes additional APCs for IL-12 production, facilitating Th1 differentiation, and can also induce the production of IFNγ by NK cells [59].

### Il-12 in MF/SS

Due to IL-12’s observed pro-inflammatory type 1 induction, it has long been studied as a potential immunotherapy for cancers, but it seems that its in vitro antitumor efficacy has not yet been well-established in humans [157].

Even if there are dated findings of a reduced or suppressed IL-12 pathway in CTCL development, especially in SS patients [60,101,158], studies regarding the impairment of the IL-12/23 axis in psoriatic patients have shown no evidence of an increased risk of altered clinical history in developing CTCLs [159]. Still, IL12/23 therapies are recent, and more long-term data are needed to confirm these findings.

Regarding the potential role of IL-12 as a treatment, there are some studies regarding the potential of the use of recombinant IL-12 in CTCLs: this is due to IL-12 being strongly able to induce Th-1 differentiation and conversion and suppressing Th-2 cytokines, making it a possible mechanism to treat MF and SS [60,138].

A clinical trial tried to assess IL-12′s role in IIA and the early stages of MF, with a 43% response in the reported patient series, but the study’s progression was interrupted due to company purchase [61].

## 14. IL-13

Different T cell subsets and dendritic cells produce interleukin 13 (IL-13), which is crucial in inhibiting pro-inflammatory cytokine production. IL-13 shares different biological activities with IL-4 by binding the IL-4Rα subunit [160,161]. However, recent studies have demonstrated a specific and nonredundant role for IL-13 in host immunity against parasites, inflammatory airway susceptibility, and tumor progression. IL-13 has a crucial role in the immune response against gastrointestinal helminth infections, such as Nippostrongylus brasiliensis [162], and a controversial role in leishmaniasis. Noben-Trauth et al. and Alexander et al. demonstrated that IL-13 promotes disease progression in the early phases of L. major and L. mexicana infections [29,163].

On the other hand, a protective role seems to be played in the chronic phases of infection [164]. Walter et al. studied the role of IL-13 in airway hyperresponsiveness and asthma in an IL-13(−/−) murine model [62], demonstrating that mice with the deletion of IL-13 failed to develop allergen-induced airway hyper-responsiveness. On the contrary, the blockade of IL-13 was ineffective in chronic disease with airway remodeling [165]. IL-13 has recently been studied in the context of cancer immunosurveillance. IL-13 is overexpressed in gastric, pancreatic, and oesophageal cancer patients [166,167]. Interestingly, the overexpression of the IL-13Rα2 chain in gastric cancer tissue is associated with a poor prognosis after gastrectomy [168]. IL-13 plays a crucial role in disease pathogenesis in different types of lymphomas, acting as an autocrine growth factor in Hodgkin‘s lymphoma and extranodal natural killer/T cell lymphoma [63,169].

### IL-13 in MF/SS

IL-13 acts as an immunosuppressive cytokine in CTCLs. Geskin et al. speculated whether IL-13 may act as an autocrine factor in lymphoma cell proliferation through IL-13Rα1 and IL-13-Rα2 signaling. In their experiments, the American group found that the IL-13 protein was expressed in mononuclear cells in close contact with malignant cell aggregates in immunohistochemistry. In addition, a higher expression of IL-13 and its receptors within the infiltrate correlated with late stages, while in the early stages, this expression was low [122]. 

As mentioned above, numerous studies have highlighted that IL-13 and IL-4 have a synergistic effect, and their expression promotes tumor cell growth and proliferation in CTCLs. Some recent studies have shown a correlation between IL-13 and the JAK/STAT pathway [170]. Indeed, neoplastic cells can secrete mediators (including IL-13, IL-22, and oncostatin M) that activate STAT3 signaling and downregulate filaggrin and filaggrin-2 expression in human keratinocytes in vivo and in vitro. The authors hypothesized that skin barrier defects in CTCLs may be related to the production of cytokines via a JAK1/STAT3-dependent mechanism, proposing a possible JAK inhibitor therapeutic candidate, such as tofacitinib. 

Despite this theoretically promising approach, Dupilumab, an IL-4RA monoclonal antibody [36], has been demonstrated to be catastrophic in CTCL patients, as stated before [171]. Hashimoto et al. [171] analyzed the expression of IL-13 receptor α2 (IL-13RA2) in the lesional skin of an SS patient who progressed after Dupilumab administration. As a possible explanation for the disease progression, the authors proposed that, despite the action on IL-4 and IL-13RA1, IL-13 may bind to IL-13RA2, leading to the exacerbation and progression of the disease.

## 15. IL-14

Interleukin-14 (IL-14), also called Alpha-taxilinis or high-molecular-weight B-cell growth, controls B cells’ growth and proliferation. It is produced mainly by T cells and specific malignant B cells [64,172].

### IL-14 and MF/SS

IL-14 seems to be higher in effusion fluids from patients with aggressive B-cell lymphomas [173] and in murine models related to the development of solid cancers [174]. A study in mice of the directed expression of IL-14 in CD19+ B cells led to marked splenomegaly and altered spleen morphology at baseline due to the expansion of marginal zone B cells [175].

Yet, its specific role has to be determined, and there are no data related to CTCLs.

## 16. IL-15 and IL-17 

Interleukin 15 (IL-15) is produced by different types of cells, including fibroblasts, keratinocytes, monocytes, macrophages, and dendritic cells, and exerts its main activity when dimerized with the common gamma chain (γc), with the same mechanism as IL-2, IL-4, IL-7, IL-9, and IL-21 on the IL-15 receptor expressed on T, B, and NK cells. IL-15 enhances CD8 T cell cytotoxic activity, B cell differentiation and immunoglobulin synthesis, and dendritic cell maturation. IL-15 shares the same receptor as IL-2 (IL-2R/IL-15Rβ; CD122), leading to JAK1/JAK3/STAT5 pathway activation. Unlike IL-2, IL-15 is not involved in the differentiation of immunosuppressive T-regs [176]. 

The Interleukin 17 Family (IL-17) encompasses six cytokines, which are IL-17A, IL-17B, IL-17C, IL-17D, IL-17E, and IL-17F (also known as IL-25; please refer to that paragraph for more information), produced by the Th17 subset of CD4+ T cells. Among them, Il-17A is the most described in the literature. IL-17A enhances the immune response against infectious agents by inducing pro-inflammatory cytokine expression (such as TNF, IL-1, and IL-6) and the production of chemokines, metalloproteinases, and antimicrobial peptides from different types of cells, including keratinocytes, fibroblasts, and epithelial cells. Moreover, IL-17 induces the expression of ICAM-1 in keratinocytes [71]. On the other hand, chronic activity of IL-17 is associated with tumorigenesis by cancer cell proliferation, MDSC recruitment, angiogenesis, and autoimmunity [177].

### IL-15 and IL-17 in MF/SS

The first studies on IL-15 supposed that such a cytokine may be considered as a survival factor [142,178,179]. IL-15 can stimulate MF cells [67,180], and in MF samples, IL-15 is highly expressed, in contrast to what is observed in normal skin. In MF/SS, IL-15 has been implicated in the recruitment of CD4+ memory T cells to the skin, the induction of T cell proliferation, and the inhibition of apoptotic cell death [65].

Due to a higher expression in advanced CTCL stages, IL-15 was assumed to promote disease progression. However, later studies observed that IL-15 is constitutively expressed in CTCL, with no stage-related expression [181,182]. In a large cohort of patients, Willerslev-Olsen et al. [145] corroborated what had been previously reported by Leroy et al. [181] on IL-15’s heterogeneous expression. They suggested that IL-15 is constitutively expressed in MF/SS and how different IL-15 levels may be related to differences in treatment regimens and microbial infections. 

Furthermore, Willerslev-Olsen et al. [66] proposed that the net effect of IL-15 expression may depend on the specific cytokine environment and cellular composition of the skin lesions, thus playing a far more complex role in CTCL pathogenesis than initially thought. 

Indeed, IL-15 can exert an antitumor action as a growth and activation factor for non-malignant T cells. Combined with IFNa, IL-15 potentiates CD8 T cells’ and NK cells’ antitumor action and promotes the growth inhibition of malignant T cells [183,184]. On the other hand, in the literature, there is evidence that IL-15, produced by malignant T cells and/or stromal cells, promotes tumor progression via the autocrine and paracrine stimulation of malignant T cells [179]. A possible link between IL-15 and IL-17A expression via the STAT3 pathway was proposed by Dummer et al. [185]. However, Willerslev-Olsen et al. [145], who observed the presence of IL-17A after using IL-15 inhibitors, denied such a connection. In the same analysis, the Danish researchers proposed that IL1–7 may be critical in the pathogenesis of the advanced stages of CTCL [66].

Mishra et al. [69] speculated whether IL-15 might play a pivotal role in MF/SS pathogenesis, in part via the epigenetic inhibition of the transcriptional repressor, Zeb1. Zeb1 inhibition may lead to the overexpression of IL-15 and the activation of specific histone deacetylases (HDAC), opening the door to whether or not a selective inhibition of HDAC1/2 may determine a halt in disease progression. As a consequence, HDAC inhibitors may play a role in MF/SS treatment.

Several investigations have found that IL-17 stimulates angiogenesis (including lymphangiogenesis) and modulates stromal cells’ function, eventually leading to tumor progression [185,186]. The tumorigenic ability of IL-17 was the subject of a recent investigation provided by Papathemeli et al. [187]. In their research, the Greek group observed changes in IL-17 (low expression) in the mRNA levels in MF/SS samples compared to those in healthy donors. The authors speculated whether low levels of IL-17A and IL-17F in mycosis fungoides may be connected to impaired immune surveillance, thereby facilitating tumorigenesis. A specific T-helper cell subset, called TH22 cells, is responsible for IL-22, IL-10, and TNF-α production without IL-17A. 

## 17. IL-16

Interleukin 16 (IL-16) is a chemokine and a modulator of T cell activation, also implied in viral replication inhibition [151]. It is produced by several APC cells, including epidermal cells, and has shown the ability to recruit and activate many other cells expressing CD4, including monocytes, eosinophils, and DCs.

It has been extensively studied for its role in immune response; IL-16 bioactivity has also been associated with the progression of some cancers [64,70].

### IL-16 and MF/SS

IL-16′s role in most cancers is still debated, but in CTCLs, it has been proven to promote cell growth by decreasing p27(KIP1) levels, while the overexpression of the secreted IL-16 molecule induces proliferation in CTCL T cells [70]. It seems that intracellular IL-16 levels are linked to a loss of surface CD26, which occurs in the early onset of CTCL stages (with a peak to IB stage). The phenomenon seems to not be irreversible, with IL-16 levels recovered in the late stages of Sézary syndromes [188].

More data about its role in MF/SS are needed.

## 18. IL-18

Interleukin 18 (IL-18) plays a key role in IFN-γ production and in Th1 and NK cell response. IL-18 is primarily produced by dendritic cells, neutrophils, macrophages, and the epithelium, including keratinocytes. IL-18, together with IL-12 and IL-15, has a role in adaptive immunity due to its ability to induce IFN-γ production via the activation of the NF-kB and STAT-4 pathways. Without IL-12 or IL-15, IL-18 does not induce IFN-γ production, but produces IL-13 and IL-4, promoting Th2 cell differentiation [73,189,190]. 

### IL-18 in MF/SS

IL-18’s role in MF/SS has been the subject of a few studies. Bostan et al. [191] performed an immunohistochemical study on skin biopsies, including MF, pityriasis lichenoides chronica (PLC), and control cases. They demonstrated that, in all stages of MF and PLC, the levels of IL-18 expression were elevated compared to those of control cases, suggesting that the activation of the inflammasome complex and subsequent IL-18 production might play a role in MF pathogenesis [191]. 

Manfrere et al. [74] studied the levels of inflammasome components, including IL-18, in SS patients. The IL-18 levels were lower in the epidermis and elevated in the dermis of SS patients compared to the control group (composed of healthy donors and patients with idiopathic erythroderma). In SS lymph nodes, IL-18 expression was elevated compared to controls. Similarly, in SS serum, IL-18 and the endogenous inhibitor IL-18-binding protein were elevated in the serum of SS patients. Interestingly, higher IL-18 levels were correlated with a reduced expression of IL-1B. The authors hypothesized that this imbalanced IL-1B and IL-18 expression in SS patients might represent a distinct inflammasome activation pathway leading to tumoral escape to apoptosis [74]. 

## 19. IL-19, IL-20, IL-22, IL-24, and IL-26

Interleukins 19, 20, 22 24, and 26 (IL-19, IL-20, IL-22, IL-24, and IL-26) are members of the IL-20 subfamily, which is part of the IL-10 family. These cytokines mainly exert anti-inflammatory and immunosuppressive activities [53,75]. 

IL-19 regulates T cells’ activity and the production of IL-4, IL-5, IL-10, and IL-13. It is involved in autoimmune and inflammatory diseases such as psoriasis and IBD. IL-19 has a controversial role in monocyte activity, inducing IL-10 production in human monocytes with the M2 phenotype, whereas it induced IL-6 and TNF-α expression in mouse M1 monocytes [192].

IL-20 is involved in the pathogenesis of inflammatory diseases, such as psoriasis, rheumatoid arthritis, and atherosclerosis [193]. Moreover, it is associated with breast cancer cell survival and bladder cancer cell migration [194,195]. 

IL-22 is a member of the IL-10 family and is produced by NK and CD4 T cells. It is considered as a cancer-promoting cytokine. Indeed, IL-22, after binding IL-22R, leads to STAT3 pathway activation and is related to the progression of different neoplasms, including pancreatic, colon, breast, and lung cancers [115].

IL-24 was originally identified as a tumor-suppressing protein named melanoma differentiation-associated 7 (MDA-7) [196]. Interestingly, IL-24 has unique antitumor activity in lung, breast, and colorectal cancer models [197].

IL-26 was initially named AK155. It is produced by epithelial cells, macrophages, NK cells, Th1, and Th17 cells, and it exerts a pro-inflammatory activity in infections and autoimmune responses [198]. 

### IL-19, IL-20, IL-22, IL-24, and IL-26 in MF/SS

IL-19 expression has been investigated in the advanced stages of CTCL. Senda et al. demonstrated that IL-19 levels were correlated positively with HMGB1, a protein associated with angiogenesis, Th2 polarization, and CTCL progression [76].

Regarding IL-22, it was studied as being related to IL-17A by a Greek group [124]. IL-22 had a relatively high mRNA expression compared to IL-17 MF/SS samples. For the group, the upregulation of IL-22 could play a role in establishing the tumor microenvironment in mycosis fungoides. IL-22 also seems to play an essential role in keratinocyte proliferation, leading to epidermal hyperplasia. A specific T-helper cell subset, called TH22 cells, is responsible for IL-22, IL-10, and TNF-α production without IL-17A. Serum IL-22 levels are positively correlated with serum sIL-2R, LDH, and CCL27 levels, and all the molecules mentioned above are known to be related to disease activity in CTCLs [125,126]. Several reports present in the literature have reported that high levels of IL-22, IL-10, and CCL20 in serum patients are correlated with the advanced stages of CTCLs [127]. Furthermore, it has been proposed that high levels of IL-22, IL-10, and CCL20 may determine, as a cascade event, the overexpression of IL-22–STAT3–CCL20–CCR6 thought to be associated with a tumor’s ability to spread to the lymph nodes and internal organs [128,129].

No data are currently available about the roles of IL-20, IL-24, and IL-26 in MF/SS.

## 20. IL-21

Interleukin-21 (IL-21) exerts its main activity when dimerized with the common gamma chain (γc), with the exact mechanism of IL-2, IL-4, IL-7, IL-9, and IL-15 on the IL-21 receptor expressed on T, B, and NK cells. Its function is to regulate cell proliferation and activity. It is primarily produced by a subset of T cells known as T follicular helper (Tfh) cells [199].

### IL-21 in MF/SS

Regarding its role in tumor genesis, IL-21 has been proven to induce cytotoxic reactions against tumors such as melanoma [199,200] and has been proven to be produced by Hodgkin’s lymphoma (HL) cancer cells (in contrast to what is known in the unaffected healthy human body), making it considered as a possible biomarker [201].

## 21. IL-22

See the paragraphs on Section 19.

## 22. IL-23

Interleukin 23 (IL-23) is an inflammatory heterodimeric cytokine composed of subunits IL-12B (in common with IL-12) and IL-23A [79]. Its structure makes it related to IL-12 and included in the heterodimeric cytokines family with Il-12, IL-27, and IL-35 [202].

It is primarily produced by activated antigen-presenting cells (APCs), such as dendritic cells, macrophages, or monocytes, but other immune cells, such as innate lymphoid cells and γδ T cells, can secrete it.

It is vital for T helper type 17 cell (Th17 cell) maintenance and expansion. Polarization to a Th17 phenotype is triggered by IL-6 and TGF-β, which activate the Th17 transcription factor RORγt [79].

### IL-23 in MF/SS

Before IL-23 was discovered, IL-12 was thought to be one of the principal causes of inflammatory disease and autoimmunity [203], but after the work of Oppmann et al. [204], a new biological model showed how IL-23 was mainly driven in psoriasis, arthritis, and immuno-bowel diseases.

This led to multiple studies on targeting it and developing drugs such as ustekinumab, guselkumab, risankizumab, and tildrakizumab [205,206].

However, as for IL-12, due to the recent discovery and lack of data, IL-23 in CTCLs is poorly understood. The work of Sugaya et al. [207] showed how CD163+ receptors are related to M2-activated macrophages, which, in contrast to classically activated ones, are not driven by IL-23 and are increased in CTCL lesional skin. 

Moreover, as for IL-12 cytokines, a study indicated no evidence of CTCL relation with IL-23 biological therapies [159], despite some case reports [208] and small patient series [209].

Actually, there are no data about the use of IL-23 biological drugs specifically for CTCLs.

## 23. IL-24 

See the paragraphs on Section 19.

## 24. IL-25

Interleukin 25 (IL-25) belongs to the IL-17 cytokine family and is also known as IL-17E. IL-25 is mainly produced by dendritic cells and exerts its activity on various types of cells, including Th cells. IL-25 leads to Th2 phenotype polarization and IL-4, IL-5, and IL-13 production. Moreover, IL-25 inhibits TH1 and TH17 responses by inhibiting IL-12 and IL-23, respectively. IL-25 is mainly involved in autoimmune diseases, allergic disorders, and parasitic infections, but its role in different types of cancers has recently come under investigation [80,210].

### IL-25 in MF/SS

IL-25’s role in MF/SS has been investigated. Nakajima et al. demonstrated that IL-25 expression was higher in MF and SS epidermal keratinocytes than in normal controls. IL-25 production is stimulated by IL-4 and IL-13 cytokines—produced by tumor cells and Th2 cells—and periostin produced by dermal fibroblasts. Moreover, the IL-25 levels in skin lesions and sera are correlated with disease progression, and IL-25 serum levels are correlated with LDH levels. IL-25 enhanced IL-13 production from tumor cells via STAT6 signaling pathways, resulting in the augmentation of a Th2-dominant microenvironment [211].

## 25. IL-26

See the paragraphs on Section 19.

## 26. IL-27 

Interleukin 27 (IL-27) is a member of the IL-6/IL-12 family and regulates the immune response. IL-27 promotes Th1 immunity and IFN-γ production by NK and T cells [212] and inhibits Th2 response [82,190]. IL-27 seems to play a controversial role in cancer immunity. IL-27 promotes antitumor immunity by NK, NKT, and CD8+ T cells [213,214]. On the other hand, IL-27Rα expression in different tumor cell lines is associated with the inhibition of effector responses and the promotion of tumor growth [215]. 

### IL-27 in MF/SS

Miyagaki et al. analyzed the serum levels of IL-27 in MF and SS patients. They observed that the IL-27 levels were higher in advanced stages compared to early stages or controls. However, the IL-27 serum level of patients with advanced-stage CTCL was inversely correlated with the number of eosinophils in peripheral blood. The authors hypothesized that IL-27 might be produced by microenvironment cells as a response to neoplastic cells to reduce Th2 immunity, typically associated with CTCL progression [129].

## 27. IL-28 and IL-29

Interleukin-28 (IL-28) presents two isoforms, IL-28A and IL-28B, belonging to the amino acid sequence and functions of the type III interferon family of cytokines, which are highly similar (in amino acid sequence) to IL-29 [54,97].

For their descriptions, refer to the paragraph on interferons.

## 28. IL-30

Interleukin-30 (IL-30) is a component of the IL-27 cytokine family, often associated with modulating immune responses. IL-30 is known for its potential role in regulating inflammation and immune reactions. However, its specific physiological functions are still being investigated [81].

### IL-30 in MF/SS

To date, IL-30 has not been extensively studied or linked explicitly to MF. However, IL-30’s physiological role suggests a potential involvement in modulating immune responses and inflammation, which could have implications for various skin conditions.

## 29. IL-31 and IL-8

Interleukin 31 (IL-31) is mainly produced after IL-4 stimulation by CD4+ Th2 helper cells, mast cells, and dendritic cells and exerts its activity on fibroblasts and eosinophils. IL-31 binds the IL-31 receptor (IL-31R), leading to JAK1/JAK2 and STAT3 signaling. IL-31 has a prominent role in pruritus pathogenesis in atopic dermatitis, prurigo nodularis, and CTCL [30,216,217]. 

Interleukin 8 (IL-8), also called CXCL8, is a chemokine produced by macrophages, epithelial cells, and airway smooth muscle cells. IL-8 is a chemotactic factor for neutrophils and other granulocytes. IL-8 exerts its oncogenic role by binding the IL-8 R localized on cancer cells and microenvironment cells (including TAM and neutrophils), leading to neo-angiogenesis and an enhancement in metastatic potential in pancreatic and bladder cancer [218,219]. 

### IL-31 and IL-8 in MF/SS

Several studies have investigated the pathogenetic role of IL-31 in CTCL [85,125,220]. IL-31 is secreted by Th-2 cells after IL-4 stimulation [221] and by malignant T cells [83]. In CTCL patients, increased IL-31 levels have been detected in both sera and skin lesions, and seem to be correlated with increased pruritus [216]. However, no univocal correlation has been established between IL-31 levels and disease progression or pruritus severity [83,220,222]. 

IL-8 is a chemotactic cytokine that is not directly involved in the pathogenesis of pruritus in CTCL [47]. However, several studies have described high IL-8 levels in pruritic dermatitis, such as atopic dermatitis [48]. In a study by Abreu et al. [49], IL-8 serum levels were similar in MF patients and healthy controls, suggesting that IL-8, per se, is not a key mediator of itch in MF/SS patients. However, owing to IL-8′s chemotactic action for neutrophils, one can speculate whether IL-8 may take part in exacerbating itch by recruiting and activating neutrophil cells. Kadin et al. [42] also proposed that high IL-8 levels may be a marker of worsened clinical outcomes in MF. Indeed, the American group found that IL-8 serum levels were correlated with CD30 serum levels. As a consequence, IL-8 may be secreted by CD30+ neoplastic cells, causing a general state of immune activation predisposing to MF progression [223]. Hence, IL-8 should be regarded as a marker of MF progression.

## 30. IL-32

Interleukin 32 (IL-32) is encoded by one gene, but has nine different isoforms because of the alternative mRNA splicing [224,225]. 

The most studied isoform is IL-32γ, and it does not belong to any cytokine family because it has almost no similar structure nor function to other cytokines.

Some isoforms seem to be related and are found in cancer cells [226,227], fostering tumor progression stimulating (NF-κB)-mediated cytokines and metalloproteinase production, as well as the stimulation of differentiation into immunosuppressive cell types in some cancer types [228]. For example, IL-32γ and IL-32β expression are associated with increased cancer cell death in colon cancer and melanoma, whereas the expression of these isoforms is associated with increased invasion and migration in breast cancer cells [224,229]. 

Also, the IL-32 isoforms α, β, and γ also play essential roles in regulating the anti-tumor immune response [230].

### IL-32 in MF/SS

Van Kester et al.’s study showed how MF may be induced by IL-32-producing cells [231], while other groups showed IL-32 to have a progressive role as an independent factor unrelated to Th2 differentiation and autocrine stimulating factor in MF and SS cells in lesional skin [232,233]. IL-32 mRNA levels have been found to increase in tumoral samples, and, when cultured, tumor viability has been impaired by adding anti-IL-32 antibodies, confirming the potential effect of IL-32 on MF and SS viability and progression [86,232].

IL-32 was recently discovered, and there are no data regarding a potential targeted therapy such as humanized immunoglobulins for cancer or other diseases.

## 31. IL-33

Interleukin-33 (IL-33) is a member of the IL-1 family and acts like an alarmin because it induces an inflammatory response after skin damage. By binding to its receptor ST2, IL-33 can polarize macrophages into M2 macrophages, induce the maturation of dendritic cells, and promote Th1-mediated responses, including cell-mediated cytotoxicity [87,234] [Kurowska-Stolarska M J Immunol 2009; Liew FY Nat Rev Immunol 2016]. IL-33/ST2 signaling in sensory neurons mediates pruritus, a symptom shared by many pathological conditions of the skin. IL-33 is highly expressed in the skin and increases in inflammatory skin lesions in atopic dermatitis, psoriasis, and scleroderma [235]. In atopic dermatitis patients, high IL-33 serum levels are positively correlated with the severity of atopic dermatitis [235]. IL-33 is also implicated in allergy and asthma development [88]. IL-33 is also thought to be related to the promotion of local progression and metastasizing of myeloproliferative neoplasms and gastric, colon, and breast cancer [236]. 

### IL-33 in MF/SS

Rustowska-Rogowska et al. [237] proposed that IL-33/ST2 signaling may be implicated in the pathogenesis of MF. Although MF patients had similar serum IL-33 concentrations as controls and IL-33 concentrations were unrelated to the MF stage, the authors hypothesised that high IL-33 levels may be confined to the skin and rapidly degraded by proteasomes. Hence, IL-33 levels may appear normal at the blood level, but not reveal increased IL-33 production in the skin of MF patients [89]. Therefore, IL-33 might accelerate MF progression via a paracrine action in the tumor microenvironment, like in patients with myeloproliferative syndromes [90]. Rustowska-Rogowska et al.’s [237] conclusion was that further studies should investigate IL-33’s expression in lesional skin.

## 32. TNF-α

TNF-α is a naturally occurring cytokine involved in normal inflammatory and immune responses. It plays an essential role in the pathogenesis of various hematologic malignancies, such as multiple myeloma, myelodysplastic syndrome, acute myelogenous leukaemia, lymphoproliferative disorders, and conditions such as graft-versus-host disease [238,239].

### TNF-α in MF/SS

Tumor necrosis factor alpha (TNF–α) has been implicated in the development of CTCL by the promotion of epidermotropism via the induction of interferon-inducible protein (IP-10) [238,240]. In addition, TNF-a acts as an autocrine growth factor, enhancing its tumorigenic action and empowering the NF-κB pathway [241]. In cell lines, anti-TNF-α antibodies downregulate CTCL cell growth, as well as NF-κB overactivation. As a result, TNF-a plays a complex role in CTCL pathogenesis along with other cytokines, such as IL-7, IL-12, and 15, by allowing neoplastic cells to gain advantages against immune system antitumor action. TNF-a is also overexpressed in psoriasis, creating a link in etiologic mechanisms between psoriasis and CTCL, particularly a defect in the mechanisms involved in inducing cell death. The availability of anti-TNF-a drugs in psoriasis has given some hope in CTCL treatment. To date, no conclusions can be drawn in patients treated with anti-TNF- α with a misdiagnosed or concomitant MF/SS, owing to the presence in the literature of MF/SS cases unmasked by the use of anti-TNF-α, cases that progressed after biological treatment administration, and cases without clinical progression after anti-TNF-α treatment [242,243,244,245,246,247].

However, extreme caution before starting anti-TNF-α treatment, as well as supplementary biopsies in case of worsening after the beginning of the treatment, has been suggested by all the authors. 

## 33. EGF

Epidermal Growth Factor (EGF) is a polypeptide produced in different tissues and was first discovered in the mouse salivary gland, but in the human body, it can be found in platelets. It is also in the urine, saliva, milk, tears, and blood plasma [248,249]. It can also be found in the submandibular glands and the parotid gland. Its mediator and receptors are modulated by cellular stress and injury mediators (such as TNF-α) [91], hormones such as estrogens [92], other growth factors (e.g., TGF-β) [250]), and environmental factors. 

It is studied for causing epidermal proliferation, modulating cellular activities, cell survival, and general tissue repair [248]. EGF’s biological activities depend upon its binding to a specific cell membrane receptor, which leads to a cell cycle progression effect on most epithelial tissues, fibroblasts, and endothelial cells [251].

### EGF in MF/SS

Only one article in the literature described a hypothetical but unproven therapeutic relationship between EGF and CTCLs, which claimed that MF develops in tissues with IFN type 1 deficiency. Since EGF has been proven to impair IFN production, it is speculated that reducing EFG functions may alter disease progression [95].

## 34. FGF

Fibroblast growth factors (FGF) regulate a broad spectrum of biological functions, including cellular proliferation, survival, migration, and differentiation. Alterations in FGF–FGF receptor expression, localization, and signaling have been implicated in several pathological processes, including malignant transformation, tumor spread, and metastasis [93,252]. 

### FGF in MF/SS

Little is known about the role of FGF in MF and SS. Queen et al. hypothesized the roles of FGF and TGF-β in paraneoplastic scleroderma pathogenesis in a patient affected by MF with CD30+ large cell transformation. FGF and TGF-β stimulate the differentiation of mesenchymal stem cells into myofibroblasts, leading to fibrotic pathway activation in MF patients [253,254]. 

## 35. PDGR

Platelet-derived growth factors (PDGFs) and their receptor (platelet-derived growth factor alpha or PDGFRα) activate the intracellular signaling pathway through tyrosine kinases (RTKs). They play critical roles in various cellular processes, particularly in cell growth, proliferation, and differentiation. Upon activation, they trigger signaling cascades that regulate cell survival, migration, and tissue repair [94,255]. Dysregulation or mutations in PDGFRα signaling have been implicated in several diseases, including certain cancers, where aberrant activation of this receptor can contribute to uncontrolled cell growth and tumor progression. PDGFRα remains a significant target for therapeutic interventions, and drugs that inhibit its activity are utilized to treat certain cancers and other conditions driven by abnormal PDGFRα signaling [256,257].

### PDGRα in MF/SS

PDGFRα is primarily known for its involvement in various cancers, especially gastrointestinal stromal tumors (GISTs), where mutations or dysregulation of PDGFRα play a significant role [139]. However, in MF/SS, the specific contribution or involvement of PDGFRα remains unexplored and far from beinb understood.

## 36. Interferon Type I, Type II, and Type III

Interferon alpha (IFN-α), interferon beta (IFN-β), which are included in the interferons type I family, interferon-gamma (IFN-γ), representing interferon type II, and the recent family of interferon type III (IFN-λ) [97] consist of cytokines produced as part of the innate immune response, and their immunomodulatory actions have been used in the treatment of several malignancies, including CTCL [258].

IFN-α is produced mainly by plasmacytoid dendritic cells (pDCs), enhancing the immune system response. Specifically, IFN- α has been found to stimulate CD8+ T cells and NK cells, thereby activating antitumor cytotoxicity [20,138,259]. Moreover, IFN- α may upregulate MHC class I expression and blunt the excess TH2 production of IL-4 and IL-5, restoring the host TH1/TH2 balance. 

IFN-β, notably regulated and synthesized by fibroblasts, is known for its antiviral activity. In the human body, IFN-β is primarily produced by fibroblasts and other immune cells, including (pDCs), in response to viral infections or other immune triggers [260]. For therapeutic purposes, recombinant IFN-β produced using biotechnological methods is mainly used to treat multiple sclerosis [261,262].

The physiological role of IFN-γ is to orchestrate cellular immunity against infections and tumor surveillance. IFN-γ enhances the antigen recognition of antigen-presenting cells (APCs) and increases the production of ROS and NOS in macrophages [96]. Moreover, IFN-γ regulates humoral immunity by B-cell proliferation and antibody class switching.

IFN-γ receptor (IFNγR) can present in two subunits, IFNγR1 and IFNγR2, that interact with JAK1 and JAK2, respectively, leading to STAT1 activation [98,263]. IFN-γ plays a central role in all the phases of “cancer immunoediting” [264], showing antitumor and tumorigenic effects. In the elimination phase, IFN-γ promotes antigen presentation, CD4+ T cell polarization to Th1, and the maturation of CD8+ T cells. In the escape phase, INF-gamma induces PD-L1 up-regulation on cancerous cells, inhibiting antitumor CD8+ T cell cytotoxicity and enhancing tumor cell survival [265,266].

IFN- λ is a relatively recent family of anti-viral cytokines consisting of four molecules, which include IFN-λ1, IFN-λ2, IFN-λ3 (also known as IL29, IL28A, and IL28B, respectively), and IFN-λ4. Its function seems to be similar to the IFN type I family, although less intense [267].

### Interferon Type I, Type II, and Type III in MF/SS

IFN-α presents an antitumor action by empowering CD8+ and NK cell action against MF cells. In addition, high IFN-alpha levels decrease the Th2 cytokinetic milieu and IL-4/IL-13 overexpression. IFN-α in different formulations has been used in MF/SS treatment, but, owing to these different formulations and the absence of prospective clinical trials, no definitive conclusions on IFN-α’s efficacy can be drawn [258,268].

A brief mention is made of IFN-β, which little is known about in the field of cutaneous lymphomas. In medical databases, it has only been the subject of small studies, mainly with small and retrospective series [260,269].

IFN-γ’s role has thoroughly been investigated in MF. Sarris et al. studied the underlying mechanisms of the epidermotropism pathogenesis in CTCL and demonstrated the cytokine loop between INF-gamma, produced by CD4+ lymphocytes, and IFN -gamma inducible protein 10 (IP-10), produced by keratinocytes. The study demonstrated the pivotal role of INF-γ in CTCL pathogenesis and Pautrier’s microabcess formations. Moreover, INF-γ and IP-10 were associated with the early stages of MF with marked epidermotropism and exclusive skin involvement. On the other hand, in SS, the level of INF-γ was lower and the Th2 response was prominent [270]. Asadullah et al. demonstrated that the overexpression of EL-10 mRNA leads to MF progression by the suppression of IFN-γ production. IFN-γ a was higher in the patch stage and decreased in a stage-dependent manner in advanced stages when epidermotropism was lost [271]. Further studies corroborated such a finding and revealed that monokine induced by IFN -γ contributes to epidermotropism, providing further evidence on the role of IFN-γ in early-stage pathogenesis [99]. Sigurdsson et al. studied the expressions of IFN γ and IL-4 in dermal infiltrate in patients with inflammatory skin disease, MF, and SS, revealing a higher expression of INF-γ in inflammatory skin disease and MF than SS [28]. 

Several groups have investigated the therapeutic role of recombinant IFN-γ a in monotherapy [245,272] or in association with other therapies, such as phototherapy, bexarotene, vorinostat, and IFN-α, providing no clear-cut results due to the small sample sizes of the studies [100,273,274,275].

The literature lacks information regarding IFN- λ and its relation to CTCLs, probably due to its recent discovery and limited knowledge.

## 37. Discussion

MF/SS are fascinating diseases owing to their intrinsic nature. Indeed, each clinical presentation has a peculiar pathogenesis [276], histology [2,277,278], and stage-related treatment [279,280,281,282]. Indeed, from a histological point of view, the diseases are characterized by neoplastic T cells resembling their regular counterparts surrounded by reactive T cells along with dendritic cells, histiocytes, and macrophages. Furthermore, in a hematologic setting, it is exceptional that lymphomas warrant skin-directed therapy as a treatment, not a systemic one (concerning the early stages). However, the mechanisms involved in the progression from early indolent stages to aggressive ones are still far from being understood. Several players are involved and connected, such as gene alterations, cytokines, and microenvironment cells [11,12,72,78,283,284,285,286,287,288,289]. 

Cytokines are a potential target of treatment and response to administered therapies. Indeed, Tsai YC et al. highlighted that a switch from a Th2 to a Th1 cytokine pattern is related to a response to extracorporeal photopheresis, stressing how the interaction between neoplastic cells and the microenvironment realized by the production of different cytokine patterns is meaningful [5,19]. The administration of IFN-α, a Th1 cytokine, is a well-known efficient treatment in advanced-stage CTCL [100,272,273,274], and its therapeutic action, at least in part, is related to restoring the host’s immune system action against the disease. Other cytokines that may be potential targets have been revealed to be harmful to patients, such as the IL-4/IL-13 inhibitor Dupilumab [34,132,133,171]. As mentioned above, the administration of Dupilumanb in misdiagnosed MF/SS had the opposite action on patients, with the development of a more aggressive disease. Caution on biologic treatment should be taken, especially for cases of non-conventional atopic dermatitis or psoriasis (another disease that can be treated with biologic therapies). 

Anti-IL-17 is potentially tumorigenic in misdiagnosed MF/SS treated as “recalcitrant” psoriasis [66,185,186]. Although cases without progression have been described, anti-TNF-α treatment [231,232,233,234,235,236] should also be managed with extreme caution for the risk of developing or unmasking a CTCL. Such evidence in the literature has left the door open to the need for a clear-cut algorithm for patients eligible for biologic therapies. The restoration from a Th2 to a Th1 pattern is also one of the significant goals of the newly available drugs such as Mogamulizumab, Brentuximab Vedotin, and Chlormetine and molecules currently under clinical trial such as Pi3K inhibitors [5,127,290,291,292,293]. From the present paper reviewing the literature, it has emerged that cytokines can be a reliable marker of some tumorigenic pathway activity, such as JAK/STAT MAPK, and the suppression of those tumorigenic pathways may be monitored by analyzing cytokine composition changes in patients. The reawakening of the immune system and the empowerment of its antitumor action, along with the apoptosis of neoplastic T cells, are major targets of the currently available drugs and the candidate ones currently under investigation. In this light, interferon drugs should be regarded as the immunotherapy pioneer in MF/SS.

**Figure 1:**
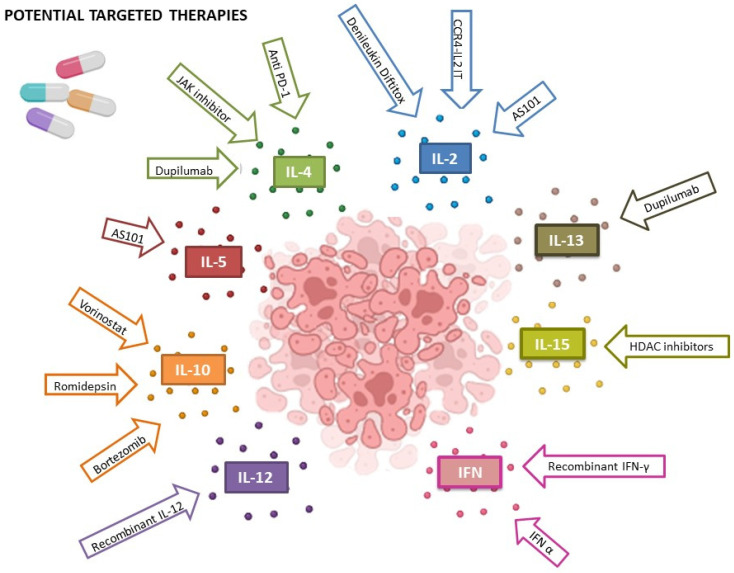
Potential and actual therapies to address CTCLs and their relative targets.

Other drugs are currently being investigated, such as Bruton’s tyrosine kinase inhibitor (Ibrutinib), which was hypothesized to decrease signaling through the T cell receptor pathway and promoting the antitumor immune response by driving selective cytotoxic Th1 CD4 effector T cell differentiation. However, a recent phase II trial [294] demonstrated limited activity on selected patients. Trials on IL-2 fusion toxin have demonstrated anti-tumor activity both as a single agent and in combination with other drugs [25,268,295]. However, the drug was not well-tolerated and its use has been limited. Due to the overexpression of the JAK3/STAT5 pathway and its cytokine expression in MF/SS [170,296,297,298], one can speculate whether JAK inhibitors may be considered as a possible MF/SS treatment. To date, limited data are available, including a case report [299] and a phase II trial [300], and no conclusions can be drawn (Figure 1).

Hypothetically, a blockade of the JAK3/STAT5 pathway should lead to the inhibition of immunosuppressive cytokines downstream. However, the lack of data demands extreme caution on JAK inhibitor administration. From the present review, it emerges that MF/SS treatment needs to be delivered at different levels, and one of them is to control the cytokine balance, with a reduction in immunosuppressive cytokines and an increase in antitumor ones.

## Figures and Tables

**Table 1:** List of described cytokines, their main mechanisms and implications in MF/SS, and their potential therapeutic roles.

Cytokine	Function	Role in MF/SS	Potential Targeted Therapies Evaluated
IL-1	Pro-inflammatory [17].	Elevated in treated patient; potential biomarker of photopheresis response [19,20].	No studies available
IL-2	Pro-inflammatory. Upregulate T cells and to increase the cytotoxicity of monocytes and natural killer (NK) [21].	May have an CTCL suppressive action, but still controversial [22,23].	AS101 inihbits IL2R and increases IL-2, suggesting an immunosuppressive role [24]. Denileukin Diftitox, which seems to reach a response of 36–40% of CTCLs in some studies [25]. CCR4-IL2 IT, in pre-clinical models seems to be promising in inducing CTCLs remission [26].
IL-3	Pluripotent and hematopoietic factor required for survival and proliferation of hematopoietic progenitor cells [27].	Unknown.	No studies available.
IL-4	Negatively modulates Th1 T cells; skews to a Th2 phenotype of naïve T cells [28].	Contributes to immune evasion and tumor progression microenvironments (still debated) [29]. Can (with IL-33) induce IL-31 secretions, involved in itch pathogenesis [28,30].	Anti PD-1 (Nivolumab) could reduce malignant Th2 cells, but it is controversial [31,32]. JAK inhibitor (Ritlecitinib) showed promising effect in reducing Th2 neoplastic cells (IIA trial ongoing) [33]. Dupilumab, by blocking IL-4 and Il-13, could induce an immune system against the tumor and blocking of Th2 cells’ proliferation, but is debated due to reported misdiagnosed MFs treated with dupilumab with a dramatic progression [34,35,36].
IL-5	Stimulates eosinophilic cascade [37].	Related to erythroderma and elevated serum levels of IgE. Seems to be overexpressed by CTCL T cells [38].	AS101 increases IFN-γ and a decreases Il-5, so to affect CTCL progression [24,39].
IL-6	Pro-inflammatory. Differentiates plasma cells and increases adhesion molecule production [40].	Hyperxpressed in CTCL samples. Seems to be related to a higher risk of MF progression [41,42]. Il-6 polymorphism may be related to worse disease prognosis [43].	No studies available.
IL-7	Hemapoietic factor stimulates the development of lymphoid lineage [44].	Linked to activation of CD8 + SS clone cells, but studies are not concordant [45,46].	No studies available.
IL-8	IL-8 is a chemotactic factor for neutrophils and other granulocytes. The oncogenic role is achieved by binding the IL-8 R localized on cancer cells and on microenvironment cells [47].	Involved in pruritus and CTCL progression [48,49].	No studies available.
IL-9	Stimulates various hematopoietic cells’ proliferation and prevents immune cells’ apoptosis. Seems to be related to hematologic neoplasias [50].	High levels have been linked in patients with SS [51] and MF [52].	No studies available.
IL-10	Anti-inflammatory. Prevention of autoimmune diseases. Can contribute to infection and tumor progression [53,54].	Higher levels of IL-10 have been detected in MF/SS biopsies compared with normal skin [55,56].	Vorinostat and romidepsin may exert their therapeutic action due to the downregulation of IL-10 RNA expression [56]. Bortezomib modulates cytokine expression in CTCL, acting on TGFβ1 and IL-10 down-regulation [57].
IL-11	Anti-inflammatory properties. Hematopoiesis, production of platelets from megakaryocytes and hemostasis [58].	Unknown.	No studies available.
IL-12	Activates NK cells and promotes the differentiation of Th1 cells [59].	Reduces or suppresses IL-12 pathway in CTCL developement, especially in SS patients [60].	Recombinant IL-12 may restore NK cells functions in CTCLs [61].
IL-13	Anti-inflammatory. Related to Th2 axis [62].	IL-13 may act as an autocrine factor in lymphoma cell proliferation through IL-13Rα1 and IL-13-Rα2 signaling [63]. A higher expression of IL-13 and its receptors correlates with late stages, while in the early stages, the expression is low [63].	Dupilumab, by blocking IL-4 and IL-13, could induce an immune system against the tumor and halt Th2 cell proliferation, but this is debated due to reported misdiagnosed MFs treated with dupilumab with a dramatic progression [34,35,36].
IL-14	Growth of B cells. Produced by T cells and certain malignant B cells [64].	Unknown.	No studies available.
IL-15	Enhances CD8 T cell cytotoxic activity, B cell differentiation, Ig synthesis, and DC maturation [65].	This is implied in the recruitment of CD4+ memory T cells to the skin, induction of T cell proliferation, and inhibition of apoptotic cell death [65,66]. Linked to later stages of CTCL and assumed to promote disease progression [67].	HDAC inhibitors [68] could halt Zeb1, which leads to an overexpression of IL-15 [69].
IL-16	Pro-inflammatory [70].	Related to early MF stages [70].	No studies available.
IL-17	Enhances immune response against infectious agents. Upregulates pro-inflammatory cytokines, chemokines, metalloproteinases, and antimicrobial peptides [71].	Low expression of IL-17 mRNA levels in MF/SS samples compared to healthy donors [66,72].	No studies available.
IL-18	Has a role in adaptive immunity. Induces IFN-γ production [73].	Higher expression in all MF stages compared to control cases. Role in tumor escape in SS [74].	No studies available.
IL-19	Pro-inflammatory [75].	IL-19 levels correlated positively with HMGB1, a protein associated with angiogenesis, Th2 polarization, and CTCL progression [76]	No studies available.
IL-20	Immunosuppression [75].	Unknown.	No studies available.
IL-21	IL-21 enhances cytotoxicity and induces the production of IFN-γ and perforin by NK cells [77].	Unknown.	No studies available.
IL-22	Immunosuppression [54].	IL-22 could play a role in establishing the tumor microenvironment in MF [78].	No studies available.
IL-23	Pro-inflammatory [79].	Unknown.	No studies available.
IL-24	Pro-inflammatory [54].	Unknown.	No studies available.
IL-25	Causes Th2 phenotype polarization and IL-4, IL-5, and IL-13 production. It inhibits TH1 and TH17 responses through inhibition of IL-12 and IL-23 [80].	Higher expression in MF and SS epidermal keratinocytes, compared to controls. IL-25 levels in skin lesions related to disease progression and serum levels correlated with LDH levels [41]. IL-25 enhances IL-13 production by tumor shifting to a Th2 dominant microenvironment [80].	No studies available.
IL-26	Pro-inflammatory [54].	Unknown.	No studies available.
IL-27	IL-27 promotes Th1 immunity, IFN-γ production by NK and T cells, and inhibits Th2 response [81].	IL-27 levels were higher in advanced stages compared to early stages or controls in MF [81,82].	No studies available.
IL-28	See IFN-γ.	Unknown.	No studies available.
IL-29	See IFN-γ.	Unknown.	No studies available.
IL-30	Pro-inflammatory [81].	Unknown.	No studies available.
IL-31	Produced by CD4+ Th2 cells, mast cells, and dendritic cells. It modulates fibroblasts and eosinophils.	Increased levels have been detected in both serum and skin lesions [83]. It seems to be related to pruritus, but is still debated [49,84,85].	No studies available.
IL-32	Immunosuppression and cancer progression [30].	IL-32 is associated with MF development and progression [86].	No studies available.
IL-33	Causes M2 macrophages’ differentiation and induces maturation of dendritic cells [87]. Promotes Th1-mediated responses, including cell-mediated cytotoxicity [88].	IL-33 might accelerate MF progression via a paracrine action in the tumor microenvironment [89,90].	No studies available.
TNF-A	Pro-inflammatory [91].	TNF–α has been implicated in the development of CTCL by the promotion of epidermotropism via the induction of interferon-inducible protein. TNF-a acts as an autocrine growth factor, enhancing its tumorigenic action and empowering the NF/KB pathway.	No studies available.
EGF	Epidermal stem cells’ proliferation and differentiation [92].	Possible role due to its known effect in inhibiting IFN type 1, low levels of which are related to MF development [91].	No studies available.
FGF	Mesenchimal stem cells’ proliferation, survival, migration, and differentiation [93].	FGF’s role has been hypothesized in paraneoplastic scleroderma in MF [93].	No studies available.
PDGFR	Varous cellular lines’ growth, proliferation, and differentiation [94].	Unknown.	No studies available.
IFN [95,96]	Pro-inflammatory [97,98,99,100].	- IFN-α presents an antitumor action by empowering CD8+ and NK cells’ action against MF cells. IFN-α reduces the Th2 cytokinetic milieu and IL-4/IL-13 overexpression [95]. - IFN-β’s role is under investigation [95]. - IFN-γ is involved in epidermotropism pathogenesis [89]. - IFN-λ’s role is unknown.	The administration of IFN -α is a well-known efficient treatment in advanced-stage CTCLS [96,97,98,99,100,101]. Recombinant IFN-γ a in monotherapy or in association with other therapies, such as phototherapy, bexarotene, vorinostat, and ifn-α, did not provide clear-cut results due to the small sample size of the studies [96,97,98,99,100,101].

## Data Availability

Data available upon request from the authors.

## Abbreviations

APC	Antigen-Presenting Cell
CD	Cluster of Differentiation
CCL	C-C Motif Chemokine Ligand
CCR	C-C Motif Chemokine Receptor
CTCL	Cutaneous Cutaneous T cell lymphoma
CXCL	C-X-C Motif Chemokine Ligand
EGF	Epdermal Growth Gactors
FGF	Fibroblast Growth Factors
JAK	JAnus Kinase
GAB	GRB2-associated-binding protein
HDAC	Histone Deacetylases
HMGB	High Mobility Group Box protein
ICAM-1	Intercellular Adhesion Molecule 1
IFN	Interferon
IL	InterLeukin
IP	Interferon-Inducible Protein
IT	ImmunoToxin
LDH	Lactate DeHydrogenase
MAPK	Mitogen-Activated Protein Kinase
MDA7	Melanoma Differentiation-Associated protein 7
MF	Mycosis fungoides
nbUVB	narrow band Ultra Violet type B
NOS	Nitrogen Oxygen Species
PBMC	Peripheral Blood Mononuclear Cell
PI3Ks	Phosphoinositide 3-kinase inhibitors
PDGF	Platelet-Derived Growth Factors
PUVA	Psoralen and Ultra Violet type A
ROS	Reactive Oxygen Species
SATB1	Special AT-rich sequence-Binding protein-1
STAT	Signal Transducer and Activator of Transcription
SS	Sézary syndrome
TAM	Tumor-Associated Macrophage
TCGF	T cell growth factor
Tfh	T follicular helper cell
TGF	Transforming Growth Factor
TNF	Tumor Necrosis Factor
Th	(Lymphocyte) T helper (Cell)
T-Regs	(lymphocyte) T-regulatory cells
NK	Natural Killer (cell)
γc	Common gamma chain
